# Designing Efficient Double RNA *trans*-Splicing Molecules for Targeted RNA Repair

**DOI:** 10.3390/ijms17101609

**Published:** 2016-09-22

**Authors:** Clemens Hüttner, Eva M. Murauer, Stefan Hainzl, Thomas Kocher, Anna Neumayer, Julia Reichelt, Johann W. Bauer, Ulrich Koller

**Affiliations:** 1 EB House Austria, Research Program for Molecular Therapy of Genodermatoses, Department of Dermatology, University Hospital of the Paracelsus Medical University Salzburg, 5020 Salzburg, Austria; e.murauer@salk.at (E.M.M.); s.hainzl@salk.at (S.H.); t.kocher@salk.at (T.K.); a.neumayer@salk.at (A.N.); j.reichelt@salk.at (J.R.); 2 Department of Dermatology, University Hospital of the Paracelsus Medical University Salzburg, 5020 Salzburg, Austria; jo.bauer@salk.at

**Keywords:** double RNA *trans*-splicing, genetic diseases, RNA therapy, type VII collagen, epidermolysis bullosa

## Abstract

RNA *trans*-splicing is a promising tool for mRNA modification in a diversity of genetic disorders. In particular, the substitution of internal exons of a gene by combining 3′ and 5′ RNA *trans*-splicing seems to be an elegant way to modify especially large pre-mRNAs. Here we discuss a robust method for designing double RNA *trans*-splicing molecules (dRTM). We demonstrate how the technique can be implemented in an endogenous setting, using *COL7A1*, the gene encoding type VII collagen, as a target. An RTM screening system was developed with the aim of testing the replacement of two internal *COL7A1* exons, harbouring a homozygous mutation, with the wild-type version. The most efficient RTMs from a pool of randomly generated variants were selected via our fluorescence-based screening system and adapted for use in an in vitro disease model system. Transduction of type VII collagen-deficient keratinocytes with the selected dRTM led to accurate replacement of two internal *COL7A1* exons resulting in a restored wild-type RNA sequence. This is the first study demonstrating specific exon replacement by double RNA *trans*-splicing within an endogenous transcript in cultured cells, corroborating the utility of this technology for mRNA repair in a variety of genetic disorders.

## 1. Introduction

RNA *trans*-splicing technology has been implemented in the treatment of a spectrum of dominant and recessive inherited genetic diseases to repair pathogenic mutations [1]. The methodology of Spliceosome Mediated RNA *trans*-splicing (SMaRT) is therapeutically used to facilitate the recombination of two distinct pre-mRNA molecules [1,2]. The introduction of only a short part of the full-length cDNA sequence of a gene into the target cells reduces unwanted side effects, common in conventional gene replacement strategies, where full-length cDNA is delivered into the cells [3]. Thus, *trans*-splicing is a favourable strategy in the treatment of monogenetic diseases, where large genes are involved. In the field of muscular dystrophies (MD), Monjaret et al. analysed the efficacy of RNA *trans*-splicing as a potential therapeutic strategy. The size of the corresponding cDNA of titin (100 kb) and dysferlin (6.2 kb), responsible for titinopathies and dysferlinopathies, respectively, exceed the packaging limits of conventional adeno-associated viral vectors. This engenders SMaRT technology as the method of choice [4]. Avale et al. generated a manipulated tau mRNA transcript in vivo by using SMaRT technology. This re-engineered tau mRNA included the missing exon 10 and displayed a corrected phenotype, which was previously blocked by mis-splicing leading to the tauopathy frontotemporal dementia and parkinsonism linked to chromosome 17 (FTDP-17) [5]. Further advantages of using SMaRT include the maintenance of endogenous regulation of the targeted gene and the possibility to reduce dominant negative protein expression [6,7,8,9,10,11]. Moreover, RNA *trans-*splicing is not only suitable to correct transcripts in inherited diseases, but can also be used in a suicide therapy approach, e.g., to induce toxin-mediated cell death in tumour cells, as demonstrated by our group and others [12,13,14].

For the severe blistering skin disease epidermolysis bullosa, we have previously described several *trans*-splicing strategies where we replaced either 5′ or 3′ regions of a transcript, in order to correct EB-associated mutations in *PLEC*, *KRT14*, *COL17A1* and *COL7A1* at pre-mRNA level [9,10,15,16,17,18]. These promising results provided us with substantial information on the optimal design of RNA *trans*-splicing molecules in order to execute a robust *trans*-splicing process. Arguably, the most elegant method of RNA repair is the combination of 3′ and 5′ RNA *trans*-splicing. This process is termed double RNA *trans*-splicing or internal exon replacement. However, due to a lack of knowledge regarding the design of an RNA *trans*-splicing molecule (RTM) and the resulting weak repair efficiency for this approach, greater effort has been invested in the development of 3′ or 5′ *trans*-splicing variants. Currently, only a few attempts were implemented for double *trans*-splicing-mediated RNA repair using minigenes [15,19]. Lorain et al. demonstrated the potential of this technology for the first time to exchange an internal exon at pre-mRNA level on the basis of the mdx dystrophin gene. Additionally, the group was able to improve the *trans*-splicing efficiency by the inclusion of an intronic splice enhancer (DISE) within the backbone of the double RTM (dRTM) [19].

In this study, we chose *COL7A1* as a target gene, in which mutations lead to the severe dystrophic variant of the skin blistering disorder epidermolysis bullosa (DEB). This disease may be inherited in a recessive (RDEB) or dominant (DDEB) form [20,21]. We have utilised our established screening system to construct a dRTM for the correction of a homozygous mutation in exon 80 (6527insC) of *COL7A1* by replacing this exon together with the downstream exon 81 by the wild-type exons. Our main aim was the selection of a dRTM that executes the splicing reaction in a coordinated manner, in order to allow normal gene expression. RNA *trans*-splicing has the auspicious feature of delivering only a short segment of the gene transcript. This results in a high transcript repair rate, and a decrease in side effects common in alternative gene therapeutic applications. We improved the safety of this technique by removing start and termination codons on the delivered dRTM, in order to avoid direct expression of shortened transcripts. Using our sophisticated fluorescence-based RTM screening system we studied the binding characteristics of randomly and rationally designed dRTMs. We were able to select a highly potent molecule (dRTM15) capable of repairing the endogenous *COL7A1* transcript in collagen type VII deficient patient cells, which sets the basis for preclinical studies to bring this promising approach into clinics.

## 2. Results

### 2.1. Screening for RNA Trans-Splicing Molecule (RTM) Binding Sites

#### 2.1.1. Screening for an Efficient Binding Domain (BD) for 3′ *trans*-Splicing Induction in a Co-Transfection Assay

**Figure 1:**
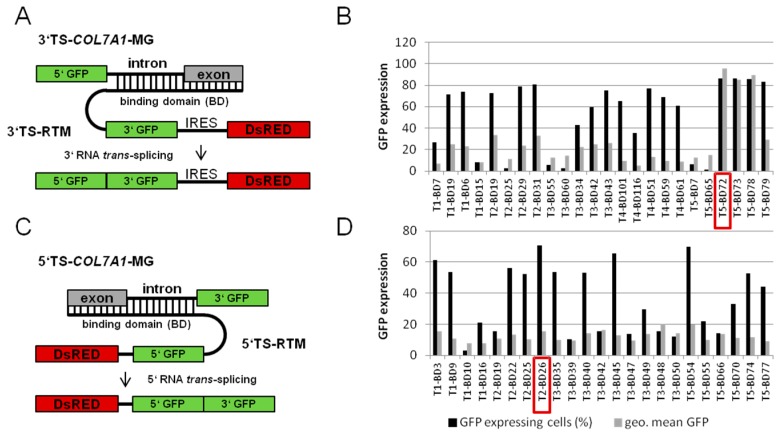
Screening for efficient binding domains. (**A**) The 3′ RNA *trans*-splicing molecules (RTM) screening system includes a minigene (MG) carrying a *COL7A1* target region, consisting of a 5′ GFP, one intron and one exon of choice (T1: intron 75/exon 76; T2: intron 76/exon 77; T3: intron 77/exon 78; T4: intron 78/exon 79; T5: intron 79/exon 80) and individual RTMs with specific antisense binding domains (BD) for the respective target region (T1–T5). The co-transfection of a *COL7A1* minigene (*COL7A1*-MG) and a functional RTM into HEK293 cells leads to the fusion of the GFP portions, provided by both pre-mRNA *trans*-splicing partners, via accurate 3′ *trans*-splicing restoring full-length GFP expression. The amount of GFP expression correlates with the level of accurate *trans*-splicing induced by the RTM; (**B**) flow cytometric analysis of RTM and *COL7A1*-MG co-transfected HEK293 cells revealed T5 as the most promising RTM binding site. Therefore BD72 (red frame in **B**), specific for intron 79, was included in dRTM design; (**C**) the 5′ RTM screening system includes MGs carrying the respective *COL7A1* target region downstream of exon 80 (T1: exon/intron 80; T2: exon/intron 81; T3: exon/intron 82; T4: exon/intron 83; T5: exon/intron 84) and individual RTMs with specific antisense BDs for the respective target region (T1–T5). The co-transfection of a *COL7A1*-MG and a functional RTM into HEK293 cells leads to the fusion of the GFP portions by accurate 5′ *trans*-splicing enabling GFP expression; (**D**) flow cytometric analysis of RTM and *COL7A1*-MG co-transfected HEK293 cells revealed target region T2 as the most promising RTM binding site. BD26 (red frame in **D**) was included in dRTM construction. The gates for FACS analysis were arranged according to the negative control (untransfected HEK293 cells) excluding all cells without GFP expression. The geometric mean (geo. mean) states the GFP intensity of the cells induced upon accurate *trans*-splicing. TS: *trans*-splicing; IRES: internal ribosomal entry site; GFP: green fluorescent protein.

Our aim was to repair the *COL7A1* transcript in a patient keratinocytes cell line by specifically replacing the mutant exon 80 with its wild-type copy through double *trans*-splicing. As a first approach, we screened for binding positions in the target pre-mRNA in order to define optimal binding domains (BDs) to be included in the double *trans*-splicing RTM (dRTM). For the design of a highly potent 3′ BD for the dRTM, we utilized a fluorescence-based screening system to identify an optimal RTM binding region upstream of exon 80 of *COL7A1* [22]. First, a pool of highly variable binding domains (BD) was generated through amplifying the *COL7A1* gene region spanning from intron 75 to exon 80 and subsequent fragmentation via sonification. The resulting BD fragments ranging from 50 to 300 nucleotides were cloned into a previously described 3′ RTM screening vector [16,17,22] (Figure 1A). In total, 24 3′ RTMs with BDs in antisense orientation to the *COL7A1* target region intron 75–exon 80 were included in co-transfection studies in HEK293 cells. Flow cytometric analysis of HEK293 cells, co-transfected with the respective *COL7A1-*minigene (*COL7A1*-MG), consisting of 5′ GFP (green fluorescent protein) and a target intron/exon region, which was either intron 75/exon 76 (T1), intron 76/exon 77 (T2), intron 77/exon 78 (T3), intron 78/exon 79 (T4), intron 79/exon 80 (T5), and individual RTMs, results in diverse GFP expression upon accurate *trans*-splicing between both molecules. The number of GFP expressing cells and the intensity of the GFP signal (geometric mean of fluorescence intensity was evaluated from all GFP positive cells) represents the functionality of the introduced RTM. Quantification of GFP expression by fluorescent-activated cell sorting (FACS) analysis identified 5′ target region 5 (intron 79–exon 80) as the most promising RTM binding site. Of all 24 analysed BDs, BD72, specific for intron 79 of *COL7A1*, was the most potent BD, capable of inducing GFP expression in over 86% of analysed cells with a high average geometric mean (Figure 1B). Thus, BD72 was selected for the construction of the 5′ part of the double RNA *trans*-splicing molecule (dRTM).

#### 2.1.2. Screening for an Efficient BD for 5′ *trans*-Splicing Induction

Similar to the screening procedure performed for the BD selection for 3′ *trans*-splicing induction a 5′ RTM library specific for the *COL7A1* target region encompassing the sequence from exon 80 to intron 84 was generated. This was achieved via PCR amplification, fragmentation and cloning of the resulting fragments into the 5′ RTM screening vector [22,23]. Functional BDs were identified by sequence analysis of a variety of individual RTM-expressing plasmids (Figure 1C). Twenty-three RTMs with antisense BD sequences were co-transfected with the respective *COL7A1*-MG representing one of the five target regions, exon/intron 80 (T1); exon/intron 81 (T2); exon/intron 82 (T3); exon/intron 83 (T4); exon/intron 84 (T5), and analysed concerning their *trans*-splicing efficiency by flow cytometric analysis. The amount of GFP expressing cells, representing accurate *trans*-splicing between the RTM and the MG, varied from 3% to 70% (Figure 1D). BD26, specific for the 5′ target region 2 (T2: *COL7A1* exon/intron 81), was identified as the most efficient BD and was therefore included in the construction of the dRTM.

The selection of an optimal 5′ BD in exon/intron 81 as a result of the pre-screening experiments led to the decision to replace both *COL7A1* exon 80 and 81 at the same time using one single dRTM.

### 2.2. Construction and Analysis of COL7A1 Specific dRTM

**Figure 2:**
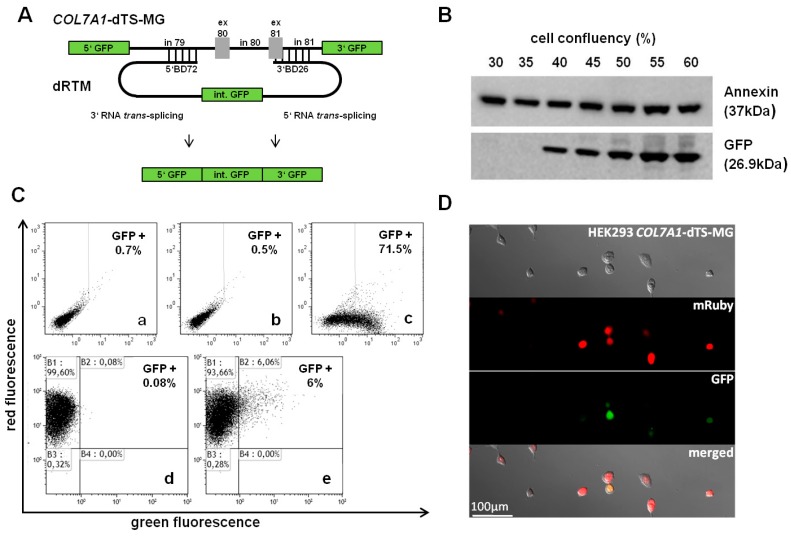
Fluorescence-based simulation of replacement of internal *COL7A1* exons. (**A**) Schematic depiction of GFP-based RTM analysis system. *COL7A1*-dTS-MG contains the 5′ and 3′ end of GFP flanking the *COL7A1* gene sequence intron 79–intron 81. dRTM-GFP comprises two binding domains specific for intron 79 and exon/intron 81, respectively, two splicing domains and the internal GFP part to be introduced into the target minigene. Accurate 5′ and 3′ *trans*-splicing between the *COL7A1*-dTS-MG and the dRTM-GFP leads to the fusion of the three individual GFP parts resulting in the expression of full-length GFP; (**B**) Restored full-length GFP was detectable at protein level by Western blot analysis of total cell extracts of HEK293 cells co-transfected with *COL7A1*-dTS-MG and dRTM-GFP. Annexin I was included as loading control; (**C**) Flow cytometric analysis of HEK293 cells transfected with *COL7A1*-dTS-MG (**a**), dRTM-GFP (**b**) or both: *COL7A1*-dTS-MG and dRTM-GFP (**c**) restoring GFP in up to 71% of all treated cells. HEK293 cells stably expressing the *COL7A1*-dTS-MG do not show GFP expression (**d**), whereas dRTM-GFP transfection leads to expression of GFP upon correct double RNA *trans*-splicing in up to 6% of all analysed cells (**e**). The FACS gates were set on the negative control, consisting of exclusively *COL7A1*-dTS-MG transfected HEK293 cells (**a**); (**D**) Microscopic analysis of HEK293 cells stably expressing *COL7A1*-dTS-MG and the red fluorescence reporter mRuby revealed the restoration of GFP expression pattern after dRTM introduction. GFP: green fluorescent protein; ex: exon; in: intron; BD: binding domain; int.: internal.

After cloning of selected BDs into the screening RTM for double RNA *trans*-splicing induction, the resulting RTMs were analysed for efficiency in co-transfection experiments in HEK293 cells and by single transfection studies in HEK293 cells stably expressing the *COL7A1*-double RNA *trans*-splicing-minigene (*COL7A1*-dTS-MG) (Figure 2A). The co-expression of the *COL7A1*-dTS-MG, adapted for the respective RTM target region (*COL7A1* intron 79–intron 81), and the designed screening dRTM containing the two selected BDs, BD72 and BD26, as well as the internal GFP sequence (dRTM-GFP) in HEK293 cells led to the production of the full-length GFP reporter upon simultaneous and accurate 3′ and 5′ *trans*-splicing (Figure 2A). Western blot analysis confirmed correct restoration of full-length GFP two days after co-transfection of *COL7A1*-dTS-MG and dRTM-GFP into HEK293 cells (Figure 2B). We observed GFP expression in over 70% of analysed cells by FACS analysis after co-transfection of both splicing constructs into HEK293 cells (Figure 2C). The transfection of the dRTM-GFP into the stable target cell line (*COL7A1*-dTS-MG) confirmed this in a simulated endogenous setting, yielding a GFP signal in up to 6% of treated cells (Figure 2C). This was further confirmed by fluorescence microscopy analysis (Figure 2D).

### 2.3. Adaption of dRTM-GFP for Endogenous Experiments

**Figure 3:**
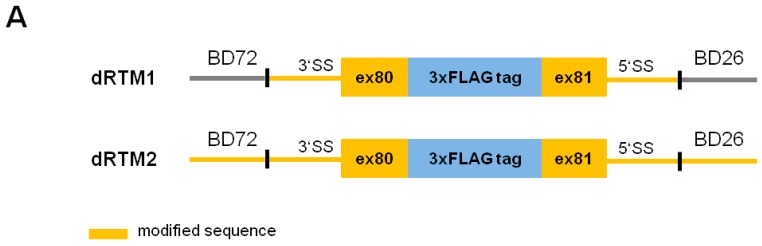

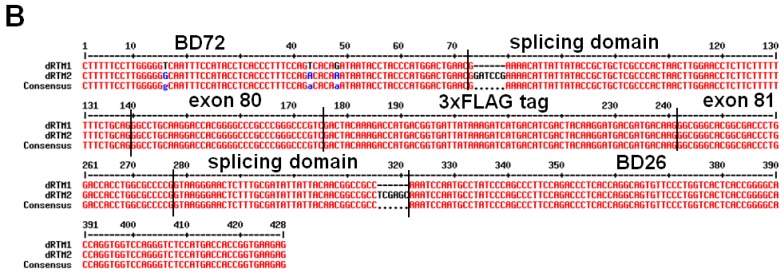
Design of endogenous dRTM1 and dRTM2. (**A**) For endogenous experiments, the internal GFP sequence of dRTM-GFP was exchanged with the wild-type coding region to introduce the *COL7A1* wild-type sequence from exons 80–81. The dRTM contains both selected binding domains (BD72 and BD26) for 3′ and 5′ *trans*-splicing induction, respectively. Possible cryptic splice sites were deleted within the coding and splicing domain (dRTM1), and the coding, splicing, and binding domains (dRTM2). Modified domains are highlighted in yellow. Additionally, a 3xFLAG tag sequence was included within the coding sequence of dRTM1 and dRTM2 to facilitate the detection of accurate RNA *trans*-splicing; (**B**) The sequence alignment was performed with the software “Multiple sequence alignment with hierarchical clustering” [24]. Black nucleotides in dRTM1: original BD72 sequence; blue nucleotides in dRTM2: modified nucleotides in BD72; black nucleotides in dRTM2: additional recognition sites for BamHI (GGATCC) and XhoI (CTCGAG).

For experiments in *COL7A1*-deficient patient cells the dRTM-GFP was modified by replacing the internal GFP portion with the wild-type sequence of *COL7A1* exon 80 and exon 81 (coding domain). In order to avoid or reduce unspecific splice events during viral dRTM packaging and expression, we removed potential cryptic splice sites within the splicing and coding domain resulting in dRTM1, and in splicing, coding and binding domains resulting in dRTM2 (Figure 3). The cryptic splice sites were predicted according to the consensus splice site sequence (donor site: A-G-cut-G-U, acceptor site: C-A-G-cut-G). In addition, both dRTMs express a 3xFLAG tag between exon 80 and exon 81 to facilitate the detection of accurate *trans*-splicing and were ordered from GeneArt Strings DNA fragments. Prior to endogenous experiments in patient keratinocytes lacking type VII collagen expression due to a *COL7A1* null mutation in exon 80, we compared the *trans*-splicing efficiency of the constructed dRTMs in the presence of the screening *COL7A1*-dTS-MG, stably expressed in HEK293 cells. Using specific primers for the detection of mRNA products maintained by accurate 3′, 5′ and double *trans*-splicing we were able to analyse the impact of the sequence alteration on the RTM′s functionality.

### 2.4. Influence of BD Variation on trans-Splicing Efficiency

**Figure 4:**
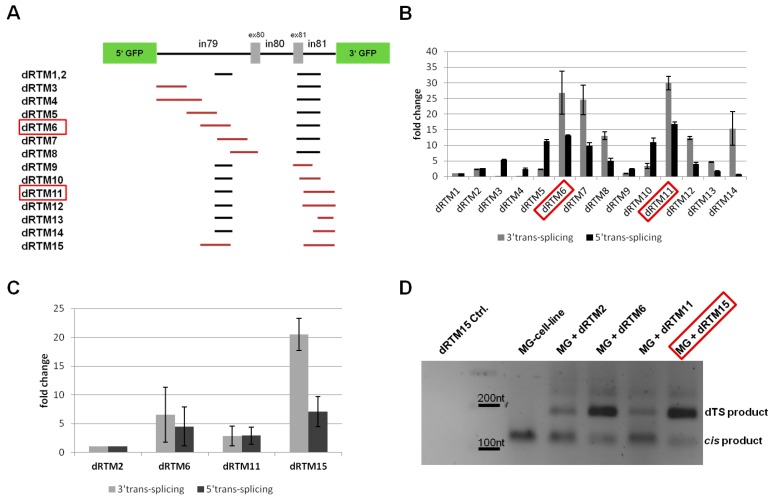
Analysis of *trans*-splicing characteristics of selected double RNA *trans*-splicing molecules (dRTMs). (**A**) Binding positions of rationally designed dRTMs specific for intron 79 and exon/intron 81 of *COL7A1*. The dRTM2-expressing vector was used as an origin vector for cloning (see Material and Methods section “Construction of dRTMs”). Black lines: original BD sequence of dRTM1,2; red lines: altered BD sequences compared to dRTM1,2; (**B**) The introduction of individual dRTMs into HEK293 cells, stably expressing the minigene (MG), results in diverse 3′ and 5′ *trans*-splicing efficiencies depending on the respective BD composition. The binding domains from dRTM6 and dRTM11 (red frames in **A** and **B**) induced the highest *trans*-splicing efficiencies and were therefore incorporated into a new dRTM, dRTM15. As reference dRTM1 was set to one; (**C**) Additionally, the combined dRTM15 was used in further transfection experiments, showing an improved 3′ and 5′ *trans*-splicing behaviour. The initial dRTM2 was used as reference and set to one; (**D**) Semiquantitative Real Time-PCR (sqRT-PCR) analysis revealed the capability of selected dRTMs, including dRTM15 (red frame), to accurately exchange the internal *COL7A1* exons 80 and 81. The product created by double RNA *trans*-splicing (5′GFP-exon80-FLAG-exon81-3′GFP) was detectable at the expected size of 173 nt and was confirmed by sequence analysis. The PCR product at the size of 107 nt was confirmed to be the product created by *cis*-splicing (5′GFP-exon80-exon81-3′GFP) within the *COL7A1*-dTS-MG (*cis* product). One of three representative PCRs is shown. dTS: double RNA *trans*-splicing.

Variations in BD-size and -position of either 5′ or 3′ BD can have an impact on the splicing capabilities of a dRTM. With the aim of further improving the existing RTM, we designed 12 new RTMs containing either a new BD for 3′ *trans*-splicing induction or a new BD for 5′ *trans*-splicing induction (Figure 4A). These BDs were cloned into dRTM2, which showed similar 5′ and 3′ *trans*-splicing efficiencies when compared to dRTM1, with the benefit of removed cryptic splice sites within the respective BD (Figure 4A). Each of the 12 new constructs consists of the original 3′ BD26 (without splice sites) and one of the new 5′ BDs (dRTM3, dRTM4, dRTM5, dRTM6, dRTM7, and dRTM8), or the original 5′ BD72 (without splice sites) and one of the newly designed 3′ BDs (dRTM9, dRTM10, dRTM11, dRTM12, dRTM13, and dRTM14) (Figure 4A). The 12 new dRTMs were individually transfected into *COL7A1*-dTS-MG-expressing HEK293 cells. This demonstrated that some of them have improved 5′ and/or 3′ *trans*-splicing rates in comparison to the initial dRTM1 and dRTM2 (Figure 4B). All selected dRTMs were capable of exchanging the target region within the *COL7A1*-dTS-MG by double RNA *trans*-splicing at the RNA level detectable by RT-PCR (Figure 4D). The experiment showed that BD variation increased the *trans*-splicing efficiency of the originally designed dRTM1 30-fold for 3′ *trans*-splicing, and 16-fold for 5′ *trans*-splicing (Figure 4B). The most efficient BDs for 3′ *trans*-splicing induction (BD of dRTM6) and 5′ *trans*-splicing induction (BD of dRTM11) were cloned into a new dRTM, termed dRTM15, with the aim of further improving the double RNA *trans*-splicing efficiency prior to endogenous experiments in RDEB patient keratinocytes (Figure 4A,C). The transcript resulting from double RNA *trans*-splicing, 5′GFP-exon80-3xFLAG-exon81-3′GFP, was amplified using specific primers for the *trans*-spliced transcript. Amplification of RNA isolated from dRTM-treated HEK293 cells stably expressing the *COL7A1*-dTS-MG, showed a PCR product at the predicted size of 173 nt of a correctly *trans*-spliced product (Figure 4D). The accuracy of *trans*-splicing was confirmed by sequence analysis. In addition to the dTS product, a competitive *cis*-spliced minigene transcript was detected at the expected size of 107 nt, which was created by *cis*-splicing within the *COL7A1*-dTS-MG. Only the most promising dRTMs 6 and 15 showed a *cis* to *trans* ratio in favour of *trans*-splicing (Figure 4D).

### 2.5. Analysis of Splicing Patterns between dRTM15 and COL7A1-MG

**Figure 5:**
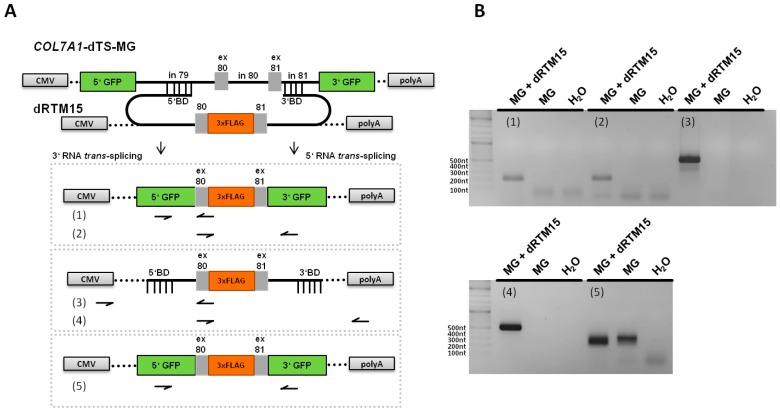
Specificity analysis between the *COL7A1*-dTS-MG and dRTM15 after transfection experiments. (**A**) Schematic representation of the performed PCRs. Five different primer combinations (1–5) were used to detect specific and possible unspecific *cis*- or/and *trans*-splicing events within or between both pre-mRNA molecules, respectively; (**B**) gel electrophoresis showed specific PCR products amplified from cDNAs derived from dRTM15/MG co- and/or exclusively MG-expressing HEK293 cells. The PCR products achieved via RT-PCRs 1 (197 nt) and 2 (220 nt) correspond to specific 3′ and 5′ *trans*-splicing products, respectively. Unspliced dRTM15 mRNA was detected via RT-PCR 3 (549 nt) and 4 (496 nt). Additionally, a specific *trans*-splicing product as well as a shortened dRTM15 PCR product (cmv-part of 5′BD) was detectable via RT-PCR 3 (faint band). RT-PCR 5 generated PCR products representing different *cis*-splicing products generated via *cis*-splicing reactions within the *COL7A1*-MG (5′GFP-3′GFP (254 nt), 5′GFP-ex80-3′GFP (290 nt), 5′GFP-ex81-3′GFP (290 nt), and 5′GFP-ex80-ex81-3′GFP (326 nt)), which were confirmed by Sanger sequencing analysis.

To analyse the specificity of the interaction between dRTM15 and the *COL7A1*-MG, different RT-PCRs were performed in order to amplify specific and potential unspecific splicing events (Figure 5A). The resulting PCR products represented specific *trans*-splicing events between both pre-mRNAs (RT-PCRs 1 and 2), and specific *cis*-splicing events within the *COL7A1*-MG (RT-PCR 5), whereby exon skipping of exon 80, exon 81 or both were detected which was confirmed by Sanger sequencing. This exon skipping likely resulted from the strong artificial splice sites at the GFP-exon 80 and exon 81-GFP borders, which were preferred for *cis*-splicing. The increased amount of detected PCR products, representing *cis*-splicing products lacking exon 80, exon 81, or both, respectively, in MG and dRTM co-transfected cells can be explained by the BD-mediated blockage of the intron 79/exon 80 and exon 81/intron 81 borders within the MG. These results indicate a partial influence of the dRTM on exon skipping within the *COL7A1* targeting region. Additionally, parts of unspliced dRTM15 were detected via RT-PCRs 3 and 4, specific *trans*-splicing products (cmv-5′GFP-exon 80) and a shortened dRTM PCR product via RT-PCR 3 (faint band: cmv-part of 5′BD), respectively (Figure 5B). The lack of detectable unwanted *trans*-splicing events of the interaction between dRTM15 and *COL7A1*-MG supports the specificity of dRTM15.

### 2.6. Detection of dRTM15 Expression

As a result of our dRTM screening, dRTM6 and dRTM15 were further developed and used for experiments in patient keratinocytes. To this end, both dRTMs were cloned into the retroviral vector pMX-IRES-Blasticidin for genomic integration into RDEB patient cells that carried the mutation in exon 80. First endogenous results were achieved using dRTM15 and hence described in this manuscript.

**Figure 6:**
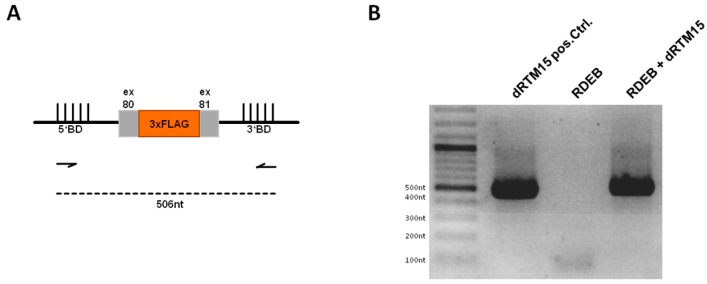
Analysis of dRTM15 expression within transduced recessive dystrophic epidermolysis bullosa (RDEB) keratinocytes. (**A**) Schematic representation of dRTM15 including both binding domains (5′ and 3′) for *trans*-splicing induction. Arrows illustrate the binding positions of the primers used for RT-PCR. The dashed line represents the expected PCR product with a size of 506 nt; (**B**) gel electrophoresis showed full-length dRTM15 expression within transduced RDEB keratinocytes. A dRTM15 expression plasmid was additionally included as PCR template representing the positive control (dRTM15 pos.Ctrl.) at the expected size of 506 nt. cDNA of untransduced RDEB keratinocytes (RDEB) served as negative control. All PCR products were confirmed by Sanger sequence analysis.

To check whether our dRTM is fully expressed in treated RDEB patient keratinocytes we performed a PCR on cDNA generated from isolated RNA (Figure 6A) using primers specific for the 5′ and 3′ binding domain, respectively. We were able to detect a specific PCR product at the expected size of 506 nt, representing the full-length dRTM15 integrated within the genome of RDEB keratinocytes (Figure 6B). The PCR product was confirmed via sequence analysis.

### 2.7. Detection of Endogenous Double Trans-Splicing at RNA Level

**Figure 7:**
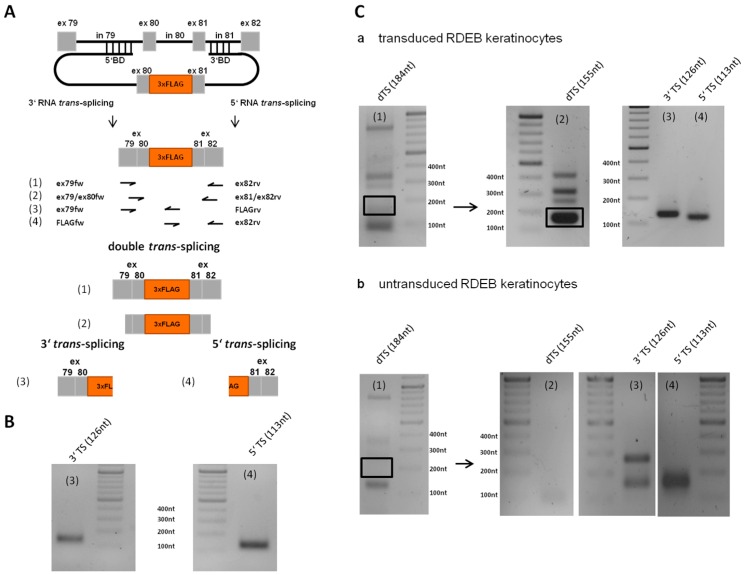
Detection of endogenous double *trans-*splicing in an RDEB patient cell line. (**A**) Schematic depiction of the endogenous double *trans*-splicing analysis system. dRTM15 contains the BDs for 3′ and 5′ *trans*-splicing induction, preselected using a *COL7A1*-MG-based dRTM screening system, and the sequence-optimized wild-type coding sequence of exon 80 and 81 interrupted by a 3xFLAG tag sequence. In order to detect accurate 3′, 5′ and double *trans*-splicing on endogenous RNA level different primer sets were used for the respective PCR amplification (1–4); (**B**) Detection of 3′ *trans*-splicing (126 nt) and 5′ *trans*-splicing (113 nt) separately. *trans*-splicing products (3, 4) were confirmed by Sanger sequence analysis; (**C**) (**a**) The expected area on the agarose gel, at which the 184 nt long double RNA *trans*-splicing product (1), was excised from the gel, purified and included as a template for subsequently performed nested PCR, revealing accurate 3′ (3), 5′ (4) and double *trans*-splicing (2) into endogenous *COL7A1* transcripts (3′ *trans*-splicing: 126 nt, 5′ *trans*-splicing: 113 nt, double *trans*-splicing: 155 nt); and (**b**) as negative control cDNA of untransduced patient keratinocytes was used as template and the PCRs were performed as preceded in (**a**). Unspecific PCR products were excised from the gel, purified and confirmed by Sanger sequence analysis as endogenous *COL7A1* sequences without RTM sequence.

RNA was isolated from dRTM15-transduced cells and RT-PCR on cDNA was performed in order to verify correct double RNA *trans*-splicing (Figure 7A). We could detect endogenous 3′ and 5′ *trans*-splicing reactions individually using different primer sets (Figure 7B). Furthermore, specific primers for exon 79 and exon 82 of *COL7A1* were used in order to amplify a 184 nt product. Nested PCR revealed a 155 nt double *trans*-spliced transcript, which was further confirmed by 3′ and 5′ *trans*-splicing reactions individually using specific primer combinations (Figure 7C). All PCR products were Sanger sequenced which confirmed accurate double *trans*-splicing, and thus the functionality of our double *trans*-splicing approach at the endogenous level.

### 2.8. Investigation of Collagen Type VII Expression Level upon trans-Splicing Induction

**Figure 8:**
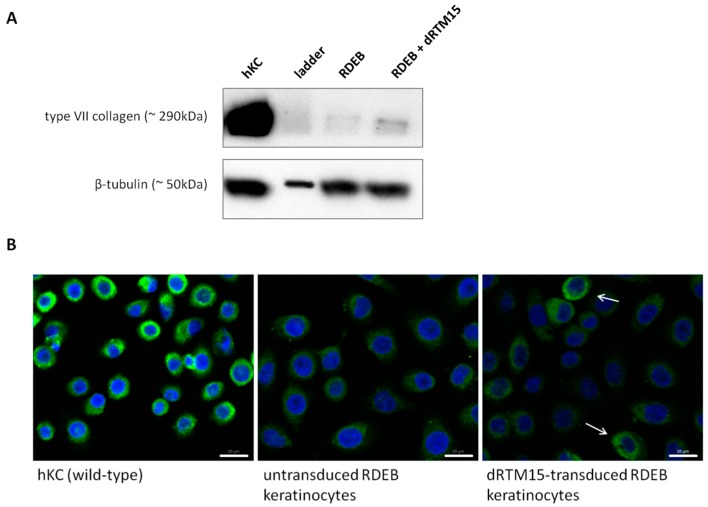
Analysis of type VII collagen expression. (**A**) Western blot analysis revealed an increase in type VII collagen expression (~290 kDa) in dRTM15-transduced RDEB keratinocytes in comparison to untransduced RDEB keratinocytes showing less type VII collagen expression. Human wild-type keratinocytes showed high type VII collagen levels and served as a control (hKC). As a protein loading control β-tubulin was used. For dRTM treated and untreated RDEB keratinocytes similar protein amounts were loaded onto the gel; (**B**) Immunofluorescense staining of wild-type keratinocytes, untransduced and RTM15-transduced patient keratinocytes. Arrows indicate dRTM15-transduced RDEB keratinocytes showing higher amounts of type VII collagen expression (green) compared to untransduced patient cells. Cell nuclei were 4′,6-diamidin-2-phenylindol (DAPI) stained (blue). hKC: human wild-type keratinocytes; RDEB: patient keratinocytes harbouring the homozygous mutation within exon 80. Scale bars represent 20 μm.

In order to determine the level of collagen type VII expression on protein level, we performed Western blot analysis using an antibody directed against the NC1 domain of type VII collagen. As a result we could detect a significant increase of type VII collagen expression in dRTM15-transduced patient keratinocytes in comparison to untreated RDEB keratinocytes in cell lysates. In the lysates of human wild-type keratinocytes (hKC) and dRTM-transduced RDEB keratinocytes (RDEB + dRTM15) a collagen type VII band at the expected size of ~290 kDa was visible compared to a faint band in untransduced RDEB cell lysate (Figure 8A). However, wild-type keratinocytes and dRTM15-transduced RDEB keratinocytes showed increased levels of type VII collagen, indicating the presence of restored proteins upon *trans*-splicing-mediated RNA repair in dRTM treated cells. Additionally, we could detect a type VII collagen restoration via immunofluorescence staining in individual dRTM15-transduced RDEB keratinocytes (Figure 8B) confirming the Western blot analysis. Compared to untransduced RDEB keratinocytes (Figure 8B, middle), respective dRTM15-transduced patient cells (Figure 8B, right panel) showed comparable type VII collagen expression levels to wild-type human keratinocytes (Figure 8B, left panel). All samples were merged with a 4′,6-diamidin-2-phenylindol (DAPI) staining.

However, an exact analysis of the repair efficiency can be performed upon FLAG tag removal within the RTM as the additional sequence may influence the functionality and folding of the protein. Additionally, the analysis of isolated single cell clones after viral dRTM transduction of patient keratinocytes will provide essential information on the general efficiency of the methodology with regard to future ex vivo or in vivo applications in mice.

## 3. Discussion

By targeting the pre-mRNA and thereby editing mRNA, SMaRT provides the opportunity to negate mutations while maintaining normal regulation of gene expression. In particular, the combination of 3′ and 5′ *trans*-splicing is an elegant and versatile way to specifically exchange single or multiple internal exon(s) of a gene of interest at pre-mRNA level. Difficulties in the design of functional double RNA *trans*-splicing molecules (dRTM) led to a dearth of knowledge and interest in the application of this strategy for RNA repair studies. Therefore very little information is found in the literature [15,19]. In general, the efficiency of RNA *trans*-splicing regardless of the applied variant is low. However, we have already shown that a low level of repaired *COL7A1* mRNA is sufficient to generate enough collagen type VII leading to full reversion of an RDEB phenotype in vitro [9,25].

The design of an efficient dRTM is more challenging, as the combined 3′ and 5′ *trans*-splicing reactions have to act simultaneously in an efficient and coordinated fashion. Here we present a reliable dRTM analysis system capable of establishing the impact of RTM such as the effect of including binding domain (BD) characteristics on efficiency. It enables analysis of the impact of BD exchange on 3′ or 5′ *trans*-splicing induction or other RTM sequence modifications on the general splicing behaviour of a given RTM. *COL7A1* has a size of over 8.5 kb when transcribed and is a suitable target for this RNA-based mutation repair. In order to specifically exchange exon 80 and exon 81 of *COL7A1*, we have sequentially designed a promising dRTM using our model system prior to subsequent endogenous applications in RDEB patient cells. Co-transfection experiments in HEK293 cells with our previously constructed screening dRTM-GFP and *COL7A1*-minigene (*COL7A1*-MG)-expressing plasmids, carrying the respective RTM binding regions, revealed the restoration of the reporter GFP in about 70% of treated cells. Transfection of the dRTM into a stable *COL7A1*-MG expressing HEK293 cell line, that was generated to simulate an endogenous setting, showed that only 6% of all cells were GFP positive. This can be explained by different expression levels of the MG due to the PiggyBac transposase-mediated integration into the target genome, which occurs randomly and often in multiple copies. In general, it is expected that the co-administration of both molecules in large amounts will positively influence the *trans*-splicing efficiency as both molecules stay in near proximity facilitating their chance to interact with the target RNA. However, the robustness and reliability of our screening system is increased by the stably expressed *COL7A1*-MG as the setting is more comparable to the endogenous situation in RDEB patient cells.

We have previously shown that the deletion of potential splice sites within the RTM sequence can decrease unwanted *cis*- or *trans*-splicing events within the RTM backbone or with other endogenous binding partners [13,17]. Therefore we modified our dRTMs after the replacement of the internal GFP portion by the coding domain (exon 80 and exon 81) in order to eliminate any potential splice sites within the RTM sequence. Studies in the stable *COL7A1*-MG expressing HEK293 cell line confirmed the functionality of the RTM after modification of the BD sequence, which was assumed would decrease the stringency of target binding. Depending upon the binding constellation of the BDs for 3′ and 5′ *trans*-splicing induction, respectively, it is obvious that one splicing reaction can be favoured at the expense of the other. The declared aim was to identify a dRTM that could perform both splicing reactions with high efficiency and specificity in order to maintain a dRTM capable of exchanging internal RNA portions in an endogenous setting. We have shown that minimal variations in the BD sequence have an enormous impact on the *trans*-splicing efficiency of constructed dRTMs approved by our single-transfection assay. The combination of the most efficient BDs for 3′ and 5′ *trans*-splicing induction results in a dRTM (dRTM15) capable of exchanging two internal exons of *COL7A1* at endogenous level. At protein level we were able to detect increased type VII collagen expression in dRTM-treated patient keratinocytes suggesting partial restoration of the protein via double RNA *trans*-splicing. Nevertheless, to fully assess the potential of this technology it is necessary to remove the 3xFLAG tag within the RTM sequence and analyse single cell clones. This will be particularly important for future in vivo experiments in mouse models.

This was the first evidence that double RNA *trans*-splicing can be a versatile tool for *COL7A1* repair. In this study we have demonstrated the comprehensible steps of dRTM design, and present challenges along the way to generate a functional RTM for endogenous mRNA repair. Using a simple screening system, we developed an efficient *COL7A1*-specific dRTM and demonstrated for the first time endogenous mRNA repair in a genetic disease model. Our data show that double RNA *trans*-splicing is an attractive method for its development for various genetic diseases.

## 4. Materials and Methods

### 4.1. Cell Culture and Transfection

For co-transfection experiments the human embryonic kidney cell line HEK293AD (Stratagene, La Jolla, CA, USA) was grown in DMEM supplemented with 10% fetal calf serum (FCS) and 100 U/mL penicillin/100 μg/mL streptomycin (Biochrom, Berlin, Germany) at 37 °C and 5% CO_2_ in a humidified incubator. Trypsin (0.05%)-EDTA (0.02%) (Biochrom, Berlin, Germany) was used for detachment of cells, and cells were pelleted by centrifugation at 350× *g* for 5 min. For transient transfection of DNA the jetPEI reagent (Polyplus-transfection SA, Illkirch, France) was used according to the manufacturer’s instructions. The transfected DNA amounts varied in the different experiments: Section 2.1: 1.5 μg target molecule + 1.5 μg RTM were transfected into HEK293 cells cultivated in a 6 well plate; Section 2.2 and Section 2.4: 3 μg *COL7A1*-MG + 3 μg dRTM were co-transfected into HEK293 cells and 5 μg of dRTM were transfected into the stably *COL7A1*-MG-expressing cell line cultivated in 60 mm plates. The RDEB patient keratinocytes carry a homozygous mutation (6527insC) in exon 80 of *COL7A1* (RDEB-TA4) [26]. This cell line was cultivated in SFM medium (Life technologies, Carlsbad, CA, USA) including the provided supplements and 100 U/mL penicillin/100 μg/mL streptomycin (Biochrom) at 37 °C and 5% CO_2_ in a humidified incubator.

### 4.2. Retroviral Transduction of RDEB Keratinocytes

Infectious retroviral particles were produced by Phoenix amphotropic cells (LGC Standards GmbH, Wesel, Germany). The cells were cultivated in DMEM (Hyclone, Perbio Science, Bezons, France) containing 10% FCII (Hyclone) and transfected by the viral plasmid DNA at a confluence of about 60% using jet-PEI transfection reagent (Polyplus-transfection) according to the manufacturer’s protocol. The medium was replaced 24 h after transfection and incubation at 37 °C and the cells were further cultivated at 32 °C. Harvesting of the supernatant containing infectious viral particles was performed from 48 h up to 96 h after transfection every 8 h and stored at 4 °C. RDEB keratinocytes were cultivated in T-25 flasks (0.8 × 10^6^ cells) and infected at a confluence of 50% with the viral suspension in the presence of 5 μg/mL of polybrene (Sigma-Aldrich, St. Louis, MO, USA) at 32 °C in humid atmosphere, and 5% CO_2_. Subsequently the flasks were centrifuged at 600× *g* for 90 min following incubation of the transduced cells over night at 32 °C. After several washing steps with Dulbecco’s PBS (Biochrom) the keratinocytes were incubated two days in cell specific medium in the presence of 25 mg/L Primocin (Invitrogen, San Diego, CA, USA). Cells were selected using Blasticidin (Invitrogen) for 8 days at a concentration of 200 mg/L.

### 4.3. COL7A1-Minigene Construction for 3′ RTM Selection

According to the protocol of Bauer et al. the *COL7A1*-minigenes (*COL7A1*-MG) were designed and cloned in order to find the optimal target region for RTM binding [15,22]. Briefly, a green fluorescence molecule (GFP) was split into two sections. The 5′ GFP part was cloned into the artificial target *COL7A1*-MG. The missing 3′ GFP part took place within the different RTMs (see Section 4.5). For the cloning procedure of the target regions different specific primer combinations were used for five different introns and the corresponding exon upstream of exon 80 of *COL7A1*.

Target region 1 (T1: intron 75/exon 76): Forward primer: 5′-GATCGATATCGATCACCCCATCCCTGCCTTAG-3′, reverse primer: 5′-GATCGCGGCCGCCTTAGCACCCTTGAGTCCAGGG-3′. Target region 2 (T2: intron 76/exon 77): Forward primer: 5′-GATCGATATCCAGTGTGTGGAATCAGCTCGGG-3′, reverse primer: 5′-GATCGCGGCCGCCCTGTCTCCTTTGGGACCTTGG-3′. Target region 3 (T3: intron 77/exon 78): Forward primer: 5′-GATCGATATCGAGGCCTCTCTCCACCCTTCC-3′, reverse primer: 5′-GATCGCGGCCGCCGGGTTGCCGTCCTGACCC-3′. Target region 4 (T4: intron 78/exon 79): Forward primer: 5′-GATCGATATCAAGTCCTTGCCCAACAGCCACAC-3′, reverse primer: 5′-GATCGCGGCCGCCGGCTTCCCTTCAGGCCCAG-3′. Target region 5 (T5: intron 79/exon 80): Forward primer: 5′-GATCGATATCGAGTGGTGGCTGAAGCACCTGG-3′, reverse primer: 5′-GATCGCGGCCGCCACTGGGCCAGGGGGGCC-3′. PCR was performed using genomic DNA from a healthy donor and GoTaq DNA polymerase (Promega, Madison, MI, USA). The PCR product was cloned into the backbone of the screening vector harbouring the 5′ split part of GFP using the restriction sites for EcoRV and NotI. Gel-extractions of amplified PCR products were performed using a GFX™ PCR DNA and Gel Band Purification Kit (GE Healthcare, Little Chalfont, UK). Plasmid preparations were carried out using a Plasmid Mini Prep Kit (Sigma-Aldrich), according to the manufacturer’s protocol. Sequence analysis of all plasmids and PCR products was performed using a 3500 ABI automated sequence analyser and ABI PRISM dye terminator cycle sequencing kit (Applied Biosystems, Waltham, MA, USA).

### 4.4. COL7A1-Minigene Construction for 5′ RTM Selection

Similar to the target constructions for the 3′ *trans*-splicing approach five different *COL7A1*-MGs were cloned into the screening vector to find the optimal target region for RTM binding. In this case, 5 introns and the respective neighboured exon downstream of exon 80 were cloned into the screening vector harbouring the 3′ split part of the GFP. For PCR amplification the following target primer combinations were designed: Target region 1 (T1: exon/intron 80): Forward primer: 5′-GATCAAGCTTCACCGGTCTGCAGGGTCCAAGAGGC-3′, reverse primer: 5′-GATCGGATCCGTTGTGGCGAAAAAGAGTCTGATGAGG-3′. Target region 2 (T2: exon/intron 81): Forward primer: 5′-GATCAAGCTTCACCGGTGGTCATGGAGACCCTGGAC-3′, reverse primer: 5′-GATCGGATCCAGAGAAAAGGGTCAAGGGCAGGG-3′. Target region 3 (T3: exon/intron 82): Forward primer: 5′-GATCAAGCTTCACCGGTCTTGCTGGCCCTGCAGGAC-3′, reverse primer: 5′-GATCGGATCCGCAAACAACCCAGAGACTGCATGAGC-3′. Target region 4 (T4: exon/intron 83): Forward primer: 5′-GATCAAGCTTCACCGGGGAGCCTGGAGAGACAGGAC-3′, reverse primer: 5′-GATCGGATCCGGAGGAAGAGAAAGTTCAGGGCAGTG-3′. Target region 5 (T5: exon/intron 84): Forward primer: 5′-GATCAAGCTTCACCGGCCTGACTGGACCTACTGGAG-3′, reverse primer: 5′-GATCGGATCCGACGGAGAACAAGTCGGATGTCAGG-3′. For cloning of the PCR products into the screening vector the restriction sites for HindIII and BamHI were used [22].

### 4.5. Construction of RTM Libraries for 3′ and 5′ trans-Splicing Induction

For BD construction we followed the protocol recently published [22]. In brief, the respective target regions (T1–T5) previously described were PCR amplified and fragmented by sonification. The resulting fragments were then cloned into the respective RTM screening vector adjacent to a spacer region, splicing domains and the corresponding part of the fluorescence molecule GFP as well as the red fluorescence reporter molecule mRuby to been able monitoring of the transfection efficiency. Individual RTMs with BDs in antisense orientation to the target region were identified by sequence analysis.

### 4.6. COL7A1 dTS-MG Construction for the Double RNA trans-Splicing Approach

The *COL7A1* dTS-minigene target plasmid was constructed by cloning of the PCR-amplified *COL7A1* target gene region intron 79/exon 80/intron 80/exon 81/intron 81 into the target screening vector, harbouring the 5′ and 3′ fragments of green fluorescent protein (*GFP*) gene, using the restriction sites for EcoRV and NotI [15,22]. For PCR amplification an intron 79 specific forward (5′-GATCGATATCGAGTGGTGGCTGAAGCACCTGG-3′) and an intron 81 specific reverse primer (5′-GATCGCGGCCGCAGAGAAAAGGGTCAAGGGCAGGG-3′) were designed. The PCR was performed using the GoTaq DNA polymerase (Promega) and as template genomic DNA isolated from an EB patient cell line with a homozygous mutation (6527insC) in exon 80 of *COL7A1* was used.

### 4.7. Construction of HEK293 Cell Line Stably Expressing the COL7A1-dTS-MG

To create a stable *COL7A1*-dTS-MG expressing cell line the MG was cloned into a PiggyBac transposon vector (System Biosciences, Mountain View, CA, USA) according to Koller et al. using the following primer pair for PCR: (forward primer: 5′-GATCTCTAGACACCATGGTGAGCAAGGGCGC-3′, reverse primer: 5′-GATCTCTAGATCACTTGTACAGCTCATCC-3′). The stable *COL7A1*-dTS-MG expressing cell line was created according to a previously published protocol [16].

### 4.8. Construction of dRTMs

For cloning of the screening dRTM for double RNA *trans*-splicing induction the selected binding domains BD72 and BD26 were combined and cloned into the screening plasmid harbouring the internal part of the GFP gene [15]. BD72 was PCR amplified using a BD72 specific forward primer (5′-GATCAAGCTTCACCCTTTTTCCTTGGGGGTCAATTTCC-3′) and a reverse primer (5′-AAGAGGTACCAGTTAGTACTCGAGCAAC-3′) specific for the spacer sequence and cloned into the RTM screening vector using the restriction sites for HindIII and KpnI. BD26 was cloned into the restriction site for NotI. For PCR amplification a BD26 specific forward primer (5′-GATCGCGGCCGCCAGATCCAATGCCTATCCCAG-3′) and a BD26 specific reverse primer 5′-GATCGCGGCCGCTCTTCACCGGTGGTCATGGA-3′) were included in the PCR.

For endogenous studies we initially used dRTM1 [24] (Figure 3), containing a FLAG tag and a sequence optimized (cryptic splice sites removed) coding region of *COL7A1*, and a dRTM (dRTMmod) without FLAG sequence, in which any cryptic donor and acceptor splice sites were removed within the binding domains. Oligos were ordered from GeneArt Strings DNA fragments (Life Technologies) and cloned into the corresponding vectors using the restriction sites for BamHI and EcoRI.

For cloning of dRTM2 [24], the modified spacer region and coding sequence including the 3xFLAG sequence from dRTM1 was PCR amplified using a specific forward primer for the 5′ spacer region (5′-GATCGGATCCGAAAACATTATTATACCGCT-3′) and a specific reverse primer for the 3′ spacer region (5′-GATCCTCGAGGCGGCCGTTGTAAT-3′). The PCR product was cloned into the screening vector pcDNA4.0 using the restriction sites for BamHI and XhoI. In a second step, the modified BD72 from dRTMmod was PCR amplified using BD72 specific forward and reverse primers (fw: 5′-GATCGGATCCCTTTTTCCTTGGGGGG-3′ and rv: 5′-GATCGGATCCGTTCAGTCCATGGGTAG-3′) and cloned into the dRTM2 vector using the restriction site for BamHI. The modified BD26 was cloned into this construct in a third cloning step using the restriction site for XhoI. For PCR amplification a BD26 specific forward (5′-GATCCTCGAGCAAATCCAATGCCTATC-3′) and reverse primer (5′-GATCCTCGAGCTCTTCCCCGGTG-3′) were included.

The replacement of the individual binding domains for 5′ *trans*-splicing and 3′ *trans*-splicing induction, respectively, was performed by PCR amplification of selected target regions and cloning of the resulting fragments in reverse complementary orientation into the dRTM2 expression vector using the restrictions sites for HindIII and BamHI (BDs for 3′ *trans*-splicing induction) or XhoI and XbaI (BDs for 5′ *trans*-splicing induction). The primer combinations used for PCR amplification are listed in Table 1.

### 4.9. Cloning of dRTM15 into Retroviral Vector

Primarily, the CMV promoter sequence was PCR amplified using a CMV-specific primer pair (fw: 5′-GATCGGATCCGACATTGATTATTGACTAGTTATTAATAGTAATCAATTACG-3′ and rv: 5′-GATCGAATTCCGGAGGCTGGATCGGTCCCGGT-3′), containing the restrictions sites for BamHI and EcoRI, and the pcDNA4.0 vector (Invitrogen) as template. The cloning of the resulting PCR fragments into the retroviral vector pMX-Blasticidin (Clontech, Mountain View, CA, USA) was performed using the restriction sites for BamHI and EcoRI. Subsequently the most promising dRTM15 was PCR amplified using BD-specific primers (fw: 5′-GATCGAATTCTTTCCTTGGGGGTCAATTTCC-3′ and rv: 5′-CTAGGCGGCCGCGAGTGACCAGGGAACACTGCCTGGTGAGGG-3′) and cloned into the retroviral vector using the restriction sites for EcoRI and NotI.

### 4.10. RNA Isolation and Cdna Synthesis

The purification of RNA from cultured cells was performed using the RNeasy Mini Kit (QIAGEN, Venlo, The Netherlands). Harvested cells were pelleted at 350× *g* and afterwards lysed in 350 μL RLT buffer containing 1% β-mercaptoethanol (Sigma-Aldrich). The following isolation steps were performed according to the manufacturer’s protocol. The RNA samples (2–3 μg) were treated with DNase I (DNase I Kit, Amplification Grade, Sigma-Aldrich) for 30 min at room temperature. The reaction was stopped by adding 1 μL of stop solution and incubation of the samples for 10 min at 70 °C. Complementary DNA synthesis was performed using the iScript cDNA Synthesis Kit (Bio-Rad, Hercules, CA, USA) according to the manufacturer’s protocol.

### 4.11. sqRT-PCR and RT-PCR

Semiquantitative Real Time-PCR was performed using a CFX96 Real Time System C1000 Thermal Cycler (Bio-Rad) and IQ SYBR Green Supermix (Bio-Rad) in order to quantify the 3′ and 5′ *trans*-splicing levels in RTM treated target cells, respectively. For PCR amplification of 3′ *trans*-splicing products a 5′ GFP specific forward primer (5′-GGGCGCCGAGCTGTTCACCGGCA-3′) and a reverse primer specific for the modified exon 80 coding sequence (5′-GGCGGGCCCCGTGGTCCTT-3′) were used for the PCR. For 5′ *trans-*splicing detection and quantification we used a forward primer specific for the modified exon 80 coding sequence (5′-GCCTGCAAGGACCACGGGGC-3′) and a 3′ GFP reverse primer (5′-CGCCGATGGGGGTATTCTGCTGG-3′). These sqRT-PCRs were performed under the following conditions: 3 min denaturation phase at 95 °C, followed by 40 cycles with 20 s denaturation at 95 °C, 20 s primer annealing at 65.6 °C, and 20 s primer extension at 72 °C. To check the specificity of the resulting PCR product, an additional melting curve analysis was performed (95 °C for 10 min, 65–95 °C in 0.5 °C increments for 5 s). For detection of the double RNA *trans*-splicing product, achieved after dRTM and *COL7A1*-dTS-MG interference on pre-mRNA level (5′GFP-exon 80-FLAG-exon 81-3′GFP), specific primers for the GFP-*COL7A1* exon boundaries (5′GFP-ex80 fw: 5′-GTGCCCTGGCCCACCCTGGCCTGC-3′ and ex81-3′GFP rv: 5′-CGGATCTTGAAGTTCACCCGGGGCG-3′) were included in the PCR. This sqRT-PCR was performed under the following conditions: 3 min denaturation at 95 °C, followed by 50 cycles with 20 s denaturation at 95 °C, 20 s primer annealing at 71.5 °C, and 20 s primer extension at 72 °C. To check the specificity of the resulting PCR product, an additional melting curve analysis was attached (95 °C for 10 min, 65–95 °C in 0.5 °C increments for 5 s).

For detection of specific and unspecific *cis*- or/and *trans*-splicing reactions within or between the *COL7A1*-dTS-MG and dRTM15, different PCRs were performed using five different specific primer combinations (listed in Table 2).

Resulting faint bands were excised and purified. The Strata Clone PCR Cloning Kit (Agilent Technologies, La Jolla, CA, USA) was used to subclone the PCR products within the StrataClone vector. The cloning procedure was performed according to the manufacturer′s protocol. Several clones were picked and analysed by Colony PCR and further Sanger sequence analysis.

To check whether our dRTM15 construct is expressed in the transduced RDEB keratinocytes a PCR was performed using primers specific for the two binding domains (5′BD fw: 5′-GATCGAATTCTTTCCTTGGGGGTCAATTTCC-3′ and 3′BD rv: 5′-CTAGGCGGCCGCGAGTGACCAGGGAACACTGCCTGGTGAGGG-3′). This PCR was performed with an annealing temperature of 60 °C and an extension time of 30 s.

For detection of accurate double RNA *trans*-splicing on endogenous level in patient keratinocytes after dRTM15 introduction, a PCR with specific *COL7A1* and FLAG primers was used. Primarily 3′ and 5′ *trans*-splicing reactions were PCR-amplified separately using a *COL7A1* exon 79 forward primer and a reverse FLAG primer for 3′ *trans*-splicing product and a FLAG forward and *COL7A1* exon 82 reverse primer for 5′ *trans*-splicing product detection. In a second step a PCR with *COL7A1*-specific primers (exon 79 fw: 5′-ATGGCTGGGCCTGAAGGGAAG-3′, exon 82 rv: 5′-GTCCTGCAGGGCCAGCAAGA-3′) and the GoTaq DNA polymerase (Promega) was performed. After excision of the expected band with a size of 184 nucleotides, representing the double RNA *trans*-splicing product *COL7A1* exon 79-exon 80-FLAG-exon 81-exon 82, from the agarose gel after gel electrophoresis, a nested PCR was performed to amplify the product for double RNA *trans*-splicing (exon 79-exon 80-FLAG-exon 81-exon 82) induction. The following primer pairs were used for PCR amplification: exon 79-exon 80-FLAG-exon 81-exon 82 (exon 79 fw: 5′-GAAGCCGGGCCTGCAAGGA-3′, exon 82 rv: 5′-CAGCAAGACCCGGGGCG-3′), exon 79-exon 80-FLAG (exon 79 fw: 5′-ATGGCTGGGCCTGAAGGGAAG-3′, FLAG rv: 5′-CTTGTCATCGTCATCCTTGTAGTCGATG-3′), FLAG-exon 81-exon 82 (FLAG fw: 5′-CCATGACGGTGATTATAAAGATCATGACATCG-3′, exon 82 rv: 5′-GTCCTGCAGGGCCAGCAAGA-3′). As negative control untransduced RDEB keratinocytes were used and the PCRs were performed as described above.

### 4.12. Analysis of GFP Expression by Flow Cytometric Analysis

Fluorescence microscopy and flow cytometric analysis were performed 2–3 days post transfection of the screening constructs into HEK293 cells. For flow cytometric analysis, the transfected HEK293 cells were trypsinised to release the cells from the culture plates, and centrifuged for 5 min at 350× *g*. The cell pellet was resuspended in 500 μL PBS and applied to the Beckman Coulter FC500. Approximately 20,000–25,000 HEK293 cells were analysed for GFP expression. The Kaluza 1.2 software (Beckman Coulter, Brea, CA, USA) was used for data analysis. Geometric mean calculations were performed using the program FlowJo (Treestar, Ashland, OR, USA).

### 4.13. Detection of trans-Splicing Products by Western Blot Analysis

Western blot analysis revealed the expression of *trans*-splicing products on protein level three days after transfection. Cell pellets were resuspended in radioimmunoprecipitation (RIPA) buffer (Santa Cruz, Dallas, TX, USA) in an appropriate volume (1 mL buffer for 2 × 10^7^ cells) and incubated for 30 min on ice. Afterwards the samples were centrifuged and the supernatants were transferred to a fresh 1.5 mL tube and stored at −20 °C.

To quantify the level of produced type VII collagen in dRTM15-transduced RDEB keratinocytes the cells were grown to confluence in conditioned medium in the presence of 50 μg/mL ascorbic acid for 72 h. Cultured cells were harvested and lyzed in 50–100 μL RIPA buffer as described above. Protein samples were mixed with 4× SDS-PAGE sample buffer (0.25 M Tris-HCl; 8% SDS; 30% glycerol; 0.02% bromphenol blue; 0.3 M β-mercaptoethanol; pH 6.8), denatured for 5 min at 95 °C and loaded onto a NuPAGE 4%–12% Bis-Tris gel (Invitrogen). Gel electrophoresis was performed using the NuPAGE MOPS SDS Running Buffer (Invitrogen) for 75 min at 150 Volt. Proteins were electro-blotted onto a nitrocellulose membrane (Amersham Hybond-ECL, Amersham, UK) at 0.25 A for 75 min. TBS-T (Tris-Base-Saline-0.1% Tween) containing 5% skimmed milk powder was used as blocking buffer (Fixmilch Instant, Maresi, Vienna, Austria).

For detection of full-length GFP the primary antibody rabbit anti-GFP IgG antibody (Biomedica, Oxford, UK) was added at a dilution of 1:500 and incubated for 90 min at room temperature. Secondary antibody was a goat-anti-rabbit HRP (DAKO, Glostrup, Denmark) used in a dilution of 1:500 for 1 h at room temperature. Luminol/enhancer and peroxide solution from the Immun-Star WesternC Chemiluminescent Kit (Bio-Rad) was used for visualization of protein bands as recommended. Anti-Annexin I mouse monoclonal IgG antibody (1:1500 in TBS-T, Santa Cruz) and a secondary HRP Envision + labeled anti mouse antibody (1:500 in TBS-Tween, DAKO) were used to detect human Annexin I which served as control for protein loading.

For type VII collagen detection the primary antibody rabbit anti-type VII collagen (kindly provided by Dr. Alexander Nyström, Department of Dermatology, University Medical Center, Freiburg, Germany) was used at a dilution of 1:3000 and incubated over night at 4 °C. The secondary antibody, anti-rabbit HRP (DAKO) was used at a dilution of 1:500 for 1 h at room temperature. A rabbit anti-beta-tubulin antibody (Abcam, Cambridge, UK) served as protein loading control in a dilution of 1:5000 incubated over night at 4 °C.

For detection and analysis a Molecular Imager^®^ ChemiDoc™ XRS system (Bio-Rad) and the Image Lab 3.0.1 software (Bio-Rad) were used.

### 4.14. Immunofluorescence Staining of type VII Collagen in Cultured Cells

Wild-type keratinocytes and both untransduced and dRTM15-transduced RDEB keratinocytes were seeded at a density of 2.08 × 10^4^ cells/cm^2^ onto glass bottom dishes (In Vitro Scientific, Mountain View, CA, USA) for immunofluorescence analysis. After 48 h, cells were fixed using 4% formaldehyde (Sigma-Aldrich) in PBS for 30 min at room temperature and afterwards permeabilized in blocking buffer (1% BSA (Sigma-Aldrich), 0.5% Triton X-100 (Sigma-Aldrich) in PBS (Invitrogen)) for 45 min at room temperature. An anti-type VII collagen antibody produced in rabbit (kindly provided by Dr. Alexander Nyström) was used as primary antibody at 1:1000 in PBS for 2 h at room temperature. As secondary antibody, Alexa Fluor 488 goat-anti-rabbit IgG (Invitrogen) was used at 1:400 in PBS (in the dark, for 1 h). The cell nuclei were stained using DAPI (Sigma-Aldrich) at 1:7000 in PBS for 10 min. Cells were analysed using an epifluorescence Zeiss Axiophot microscope (Carl Zeiss, Oberkochen, Germany).

## 5. Conclusions

Epidermolysis bullosa (EB) is an inherited cutaneous blistering disease caused by mutations located in genes encoding proteins localised to the basement membrane zone of the epidermis. Pathogenic mutations within *COL7A1* lead to the dominant or recessive dystrophic EB type (DDEB and RDEB, respectively). In this study, we used an RDEB cell line as a model system to specifically repair a mutation in exon 80 of *COL7A1* using the combined application of 3′ and 5′ RNA *trans*-splicing. To this end, we designed a retroviral vector carrying a double RNA *trans*-splicing molecule (dRTM), expressing only the short exon sequence (72 nt) to be exchanged into the endogenous transcript together with specific binding and splicing elements. The delivery of only a short portion of coding sequence facilitates viral packaging and transduction and reduces possible rearrangements caused by the highly repetitive *COL7A1* sequence. Nevertheless, the design and construction of a functional dRTM is challenging. Here we describe a reliable method for dRTM construction for therapeutic use in type VII collagen-deficient RDEB patient keratinocytes. By implementing our established RTM screening system, we were able to select a dRTM for *COL7A1* repair, highlighting the potential of this technology for therapeutic use.

## Figures and Tables

**Table 1:** Primer sets used for PCR amplification building up modified double RNA *trans*-splicing molecules (dRTMs).

dRTMs	Forward Primer	Reverse Primer
dRTM3	5′-GATCGGATCCGTGAGTGGTGGCTGAAGCACC-3′	5′-GATCAAGCTTGAGAGGCACACAGACACAGGTACAC-3′
dRTM4	5′-GATCGGATCCGTGAGTGGTGGCTGAAGCACC-3′	5′-GATCAAGCTTCACACACAGCACAGGCACACACAGGC-3′
dRTM5	5′-GATCGGATCCTGTGTGTGCCTGCTTGTGTG-3′	5′-GATCAAGCTTGGACTGCCTCTCATAGATAGCACAC-3′
dRTM6	5′-GATCGGATCCTGTGGTTGTATGTGGATGTGTGTGTGC-3′	5′-GATCAAGCTTTTTCCTTGGGGGTCAATTTCCATAC-3′
dRTM7	5′-GATCGGATCCATGGGTAGGTATTATCTGTGACTGG-3′	5′-GATCAAGCTTGGGCACTGATGAGCCTCAATCTGG-3′
dRTM8	5′-GATCGGATCCAAGCCCCCAGAGGTTGGGAACAG-3′	5-GATCAAGCTTACTGGGCCAGGGGGCCTCTTG-3′
dRTM9	5′-GATCTCTAGAGGTGGTCATGGAGACCCTG-3′	5′-GATCCTCGAGCCTTCCAGACCCTCACCAGGC-3′
dRTM10	5′-GATCTCTAGACTGGTGCCCCGGTGAGTGACCAGGGA-3′	5′-GATCCTCGAGGGCTCATCAGCTGTGGCCAATGCC-3′
dRTM11	5′-GATCTCTAGAGAGTGACCAGGGAACACTGCCTGG-3′	5′-GATCCTCGAGAAAAGGGTCAAGGGCAGGGAACAGG-3′
dRTM12	5′-GATCTCTAGAGTGAGTGACCAGGGAACACTGC-3′	5′-GATCCTCGAGCTAGAGAAAAGGGTCAAGGGCAG-3′
dRTM13	5′-GATCTCTAGACCACAGCTGATGAGCCAGGCC-3′	5′-GATCCTCGAGGGGTCAAGGGCAGGGAACAGGGC-3′
dRTM14	5′-GATCTCTAGAGCTGGGATAGGCATTGGCCACAGC-3′	5′-GATCCTCGAGGAGAAAAGGGTCAAGGGCAGGGAACAG-3′

**Table 2:** Primer combinations used for splicing pattern analysis between *COL7A1*-dTS-MG and dRTM15 (shown in Figure 5). dTS, double RNA *trans*-splicing; MG, minigene.

Combination	Forward Primer	Reverse Primer
1	5′-GGGCGCCGAGCTGTTCACCGGCA-3′	5′-GGCGGGCCCCGTGGTCCTT-3′
2	5′-GCCTGCAAGGACCACGGGGC-3′	5′-CGCCGATGGGGGTATTCTGCTGG-3′
3	5′-TGGATAGCGGTTTGACTCAC-3′	5′-GGCGGGCCCCGTGGTCCTT-3′
4	5′-GCCTGCAAGGACCACGGGGC-3′	5′-CACCCCACCCCCCAGAATAG-3′
5	5′-GGGCGCCGAGCTGTTCACCGGCA-3′	5′-CGCCGATGGGGGTATTCTGCTGG-3′
